# Stochastic Ion Channel Gating in Dendritic Neurons: Morphology Dependence and Probabilistic Synaptic Activation of Dendritic Spikes

**DOI:** 10.1371/journal.pcbi.1000886

**Published:** 2010-08-12

**Authors:** Robert C. Cannon, Cian O'Donnell, Matthew F. Nolan

**Affiliations:** 1 Textensor Limited, Edinburgh, United Kingdom; 2 Neuroinformatics Doctoral Training Centre, University of Edinburgh, Edinburgh, United Kingdom; 3 Centre for Integrative Physiology, University of Edinburgh, Edinburgh, United Kingdom; Université Paris Descartes, Centre National de la Recherche Scientifique, France

## Abstract

Neuronal activity is mediated through changes in the probability of stochastic transitions between open and closed states of ion channels. While differences in morphology define neuronal cell types and may underlie neurological disorders, very little is known about influences of stochastic ion channel gating in neurons with complex morphology. We introduce and validate new computational tools that enable efficient generation and simulation of models containing stochastic ion channels distributed across dendritic and axonal membranes. Comparison of five morphologically distinct neuronal cell types reveals that when all simulated neurons contain identical densities of stochastic ion channels, the amplitude of stochastic membrane potential fluctuations differs between cell types and depends on sub-cellular location. For typical neurons, the amplitude of membrane potential fluctuations depends on channel kinetics as well as open probability. Using a detailed model of a hippocampal CA1 pyramidal neuron, we show that when intrinsic ion channels gate stochastically, the probability of initiation of dendritic or somatic spikes by dendritic synaptic input varies continuously between zero and one, whereas when ion channels gate deterministically, the probability is either zero or one. At physiological firing rates, stochastic gating of dendritic ion channels almost completely accounts for probabilistic somatic and dendritic spikes generated by the fully stochastic model. These results suggest that the consequences of stochastic ion channel gating differ globally between neuronal cell-types and locally between neuronal compartments. Whereas dendritic neurons are often assumed to behave deterministically, our simulations suggest that a direct consequence of stochastic gating of intrinsic ion channels is that spike output may instead be a probabilistic function of patterns of synaptic input to dendrites.

## Author Summary

The activity of neurons in the brain is mediated through changes in the probability of random transitions between open and closed states of ion channels. Since differences in morphology define distinct types of neuron and may underlie neurological disorders, it is important to understand how morphology influences the functional consequences of these random transitions. However, the complexities of neuronal morphology, together with the large number of ion channels expressed by a single neuron, have made this issue difficult to explore systematically. We introduce and validate new computational tools that enable efficient generation and simulation of models containing ion channels distributed across complex neuronal morphologies. Using these tools we demonstrate that the impact of random ion channel opening depends on neuronal morphology and ion channel kinetics. We show that in a realistic model of a neuron important for navigation and memory random gating of ion channels substantially modifies responses to synaptic input. Our results suggest a new and general perspective, whereby output from a neuron is a probabilistic rather than a fixed function of synaptic input to its dendrites. These results and new tools will contribute to the understanding of how intrinsic properties of neurons influence computations carried out within the brain.

## Introduction

The appropriate level of physical detail required to understand how complex processes such as cognition and behavior emerge from more simple biological structures is unclear [1], [2]. For example, while it is possible to account for certain aspects of nervous system function using models that represent each neuron as a simple integrate and fire device, it is increasingly clear that this approach does not capture the full range of computations that many real neurons carry out [3], [4]. Dendritic and axonal morphology are defining features of neuronal cell types and have important influences on the computations that a neuron performs [5]. Differences in morphology determine how neurons respond to synaptic input and are sufficient to produce distinct patterns of spontaneous activity [6] and degrees of action potential back-propagation from the soma into the dendrites [7]. Cable theory and compartmental modeling provide a foundation for predicting the propagation of electrical signals in the dendrites and axons of neurons [8], [9]. However, while the assumption that transitions between open and closed states of ion channels can be treated as a deterministic process may be sufficient for some purposes, recent evidence suggests that stochastic transitions between the states of individual ion channels could influence computations carried out by neurons [10]–[17]. Stochastic opening and closing of ion channels causes ‘noisy’ fluctuations in the current or voltage recorded from a neuron [18]–[20]. While cable theory suggests that fluctuations of this kind might be particularly important in fine structures such as axons and dendrites [21], we nevertheless know very little about how neuronal morphology and stochastic gating of ion channels interact to determine how neurons respond to synaptic input. Given the difficulty of reducing detailed morphological models to simple analytical forms that could also incorporate stochastic gating of individual ion channels [22], experimentally constrained numerical simulations will be important to enable these issues to be explored systematically.

Investigation of stochastic ion channel gating using numerical simulations has been limited by trades-offs between simulation accuracy and computation time [22]. A simple approach is to add noise sources to deterministic models. However, as ion channels have multiple functional states with transitions that often depend on the membrane voltage [11], [15], [22], [23], this may not accurately account for the noise introduced by ion channel currents. A more accurate alternative is to explicitly model transitions between different functional states for each ion channel on a neuron's membrane. However, for neurons with complex axonal or dendritic architectures there are two substantial obstacles to this approach. First, typical central neurons express large numbers of ion channels and simulations must be repeated many times to obtain statistically valid descriptions [24]. This is a formidable computational task and even relatively straightforward simulations of the consequences of stochastic channel gating can require substantial computing time (see e.g. [12], [13]). Second, each neuronal ion channel occupies a specific location on the extra-cellular membrane, whereas most neuronal models represent the distribution of ion channels as the density of a deterministic conductance across an area of membrane. Although this formalism has been successful for simulating many aspects of neuronal activity, it is of less use for models that explore the consequences of the localization of individual ion channels, for example to evaluate the macroscopic effects of short range interactions between ion channels and other signaling molecules [25], or the consequences of spatially heterogeneous distributions of ion channels within relatively small sub-cellular structures such as dendritic spines and axon terminals [26], [27].

To address the functional consequences of stochastic ion channel gating in neurons with extensive dendritic or axonal arborizations we developed a parallel stochastic ion channel simulator (PSICS), which enables efficient simulation of the electrical activity of neurons with complex morphologies and arbitrary localization of stochastic ion channels on the extracellular membrane, while also addressing limitations of previous approaches. We have also developed an interactive tool (ICING) for visualization and development of models of neurons containing uniquely located ion channels. Here, we illustrate the use of PSICS and ICING, outline the computational strategies used and provide benchmark data for evaluation. We then identify previously unappreciated differences between the effects of stochastic ion channel gating on somatic and dendritic membrane potential activity in several different morphological classes of neuron. We show that the consequences of stochastic gating depend on dendritic morphology and suggest novel functional roles for the kinetics of ion channel gating. Using a previously well-validated realistic model of a CA1 pyramidal neuron we demonstrate that stochastic ion channel gating influences spike output in response to dendritic synaptic input. We show that stochastic gating of axonal or dendritic ion channels substantially modifies synaptically driven dendritic and axonal spike output, with stochastic gating of voltage-dependent sodium and potassium channels having the greatest impact and hyperpolarization-activated channels the least. By demonstrating that neuronal responses to dendritic synaptic input can be intrinsically probabilistic, these results offer a new and general perspective on synaptic integration by central neurons. Full documentation for PSICS/ICING as well as the software, source code and examples are available from the project website (http://www.psics.org).

## Results

### Model specification and visualization

**Figure 1:**
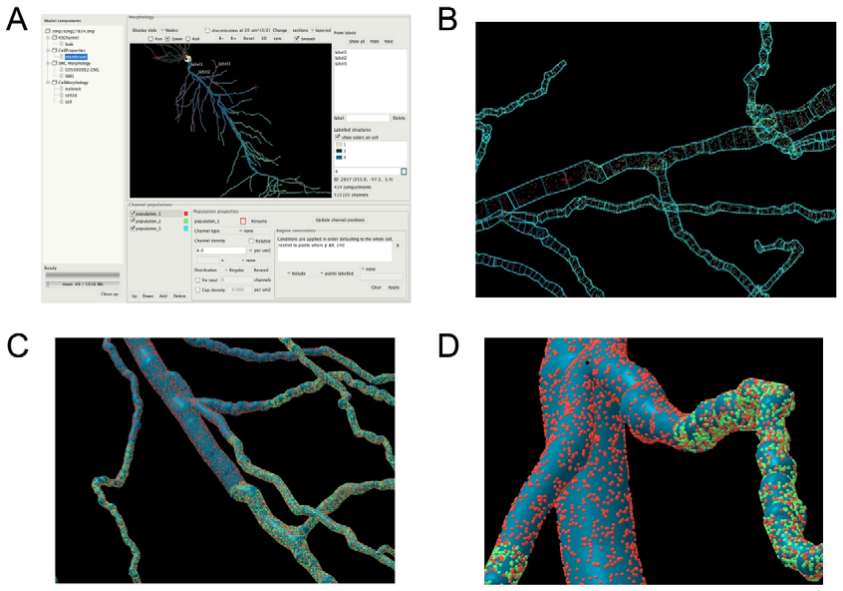
Specification and visualization of ion channel location. In PSICS individual ion channels have unique locations that can be viewed along with the compartmentalization chosen for a particular simulation using additional software called ICING. (A) Screen shot of ICING. (B) Detailed view of the compartmentalization of part of the model neuron in (A). (C) Low magnification 3 dimensional detail of dendritic branches from (B). (D) High magnification 3 dimensional detail of dendritic branches in (C) illustrating the location of individual ion channels.

To investigate the functional consequences of stochastic ion channel gating for neurons with complex dendritic or axonal morphologies, we first developed new software tools that enable accurate, fast simulation (PSICS) and visualization (ICING) of neuronal models that contain stochastically gating ion channels. The organization and development of the new software tools are described in Text S1, Figure S1 and in more detail on the project website (http://www.psics.org). Here, we briefly outline novel features of model specification and visualization, before describing key benchmark data and simulation experiments that evaluate the functional impact of stochastic ion channel gating in different neuronal cell types. The new software uses a simple XML file structure that enables components of a model either to be constructed manually, to be configured using a graphical interface (Figure 1A), or in the case of ion channels and morphologies to be imported from other programs and databases that allow saving of models in the NeuroML format. For example the morphology of the model CA1 pyramidal neuron shown in Figure 1 was downloaded as a NEURON simulation from the modelDB website (http://senselab.med.yale.edu/modeldb) and exported from NEURON as a .xml file. Similar methods can be used to import models developed with Neuroconstruct (http://www.neuroconstruct.org/) [28].

To specify the membrane conductance we adopted a new approach in which the location of each individual ion channel is first uniquely determined (Figure 1). This approach is complementary to that of the program MCell [29], which simulates movement and reactions of molecules within and around cells. In contrast, other neuronal modeling software approximates ion channel location as an average conductance density across a region of membrane. Before simulations are run in PSICS the neuron is discretized into sections that are then treated as isopotential compartments. As neurons are rarely at steady-state and have conductance that varies with membrane voltage, we implemented a discretization procedure that balances the capacitive charging rates for adjacent compartments (see Methods). The granularity of the discretization process is set by the user and determines the number of channels in a particular compartment. After discretization the PSICS simulation engine will by default compute the activity of the population of channels in an isopotential compartment, rather than the activity of each individual ion channel. Modifying the granularity of the discretization process changes the number of channels per compartment, but not the actual distribution or location of channels in the model.

Since the presently available tools for visualization and development of neuronal models are aimed primarily at deterministic simulations, we developed a graphical tool (ICING) to allow display and manipulation of neuronal models with complex three-dimensional architectures and many discrete membrane ion channels (Figure 1). ICING reads neuron morphologies specified either in NeuroML or as .swc files generated by the Neurolucida reconstruction program and used by the NeuroMorpho.org database (http://neuromorpho.org/). This enables components of a PSICS model to be visualized and edited. For example, to: 1) specify the size of compartments to use for the simulation, 2) select ion channels to be included in the model neuron, 3) select sections of the model for insertion of a particular ion channel class, 4) set rules that dictate the distribution of ion channels in their designated sections. The model neuron and its associated ion channels can be displayed in a variety of formats, for example to emphasize labeled sub-regions of the model (Figure 1A), to illustrate the compartmental boundaries in a model (Figure 1B), or to provide a detailed 3-dimensional exploration of the neuron morphology and ion channel distribution (Figure 1C–D).

### Simulation of stochastic ion channels

We represent ion channels using Markov models, in which each ion channel may be in one of a number of discrete states with the probability of transition to any other state determined independently of the channel's previous history [24], [30], [31]. To efficiently simulate stochastic transitions between states of a channel we developed a modified version of the tau leap method (see Methods) [32], [33]. The algorithm we use is equivalent to sampling an exact realization of the number of channels in a particular state at the end of each time step. In principle this results in shorter simulation times than algorithms that track the exact times of transitions between states [34], [35], or methods that permit a maximum of one transition per ion channel during each step [11], [23]. To further reduce the simulation time the algorithm considers only channels with a non-negligible probability of making a transition during a particular step (see Methods). At any particular sample time point and membrane potential, the tau leap algorithm should not produce any systematic error in the mean or variance of the current. However, the modified tau leap algorithm will not explicitly represent transitions that take place between time-points. We show below how this algorithm is particularly advantageous for current-clamp simulations in which high frequency current fluctuations are filtered by the neuronal membrane. We will also address how the choice of simulation parameters determines the accuracy and computation time.

**Figure 2:**
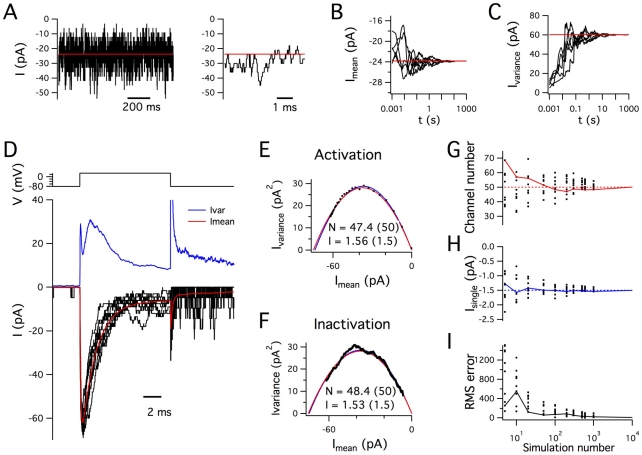
Accurate stochastic ion channel simulation. (A) Examples of simulated stochastic (black traces) and deterministic (red traces) currents in a membrane patch containing 50 stochastic Na^+^ channels with single channel conductance of 20 pS. The membrane potential is clamped at −20 mV. The expanded trace (right) shows the first 5 ms of the compressed trace (left). (B–C) Cumulative estimate of the mean (B) and variance (C) of stochastic currents measured as in (A) are plotted as a function of time. Examples from 5 separate simulations of duration 100 s are shown. Values between 100 and 1000 s are from concatenation of separate 100 s simulations. (D) Examples of 10 simulated current responses (black traces, lower plot), of the membrane patch simulated in (A–C), to a step change in membrane potential from −80 mV to +30 mV (upper plot). The mean (red trace) and variance (blue trace) are calculated from 1000 stochastic current responses. (E–F) Plot of membrane the membrane current variance as a function of the mean membrane current for the rapid activation phase (E) and slower inactivation phase (F) of the 1000 simulated current responses used to obtain the data for (D). The time window for the activation phase is 0–0.3 ms after the onset of the voltage step, whereas the time window for the deactivation phase is 0.3–10 ms after the onset of the voltage step. The number of single channels (N) and the single channel current (I) are estimated from the fit to the simulation data. The red parabola is the variance-mean relationship predicted from I and N of the model and the blue parabola is the fit to the simulation data. (G–H) Estimates for the number of channels (G) and single channel current (H), obtained by variance mean analysis of the inactivation phase of the current responses analyzed as in (D), plotted as a function of the number of simulated responses used for the analysis. Each dot corresponds to a set of data used for analysis. The continuous lines show convergence of the estimates as additional simulations are analyzed up to a maximum of 10^4^ simulated responses. (I) The RMS error, calculated from the difference between the variance mean fit and the expected variance mean relationship, is plotted as a function of the number of simulated responses. The solid lines indicate progressive convergence up to a maximum of 10^4^ simulated responses. For all examples the simulation time step was 10 µS.

To first evaluate the modified tau leap algorithm for stochastic simulations we consider a simple three-state Na^+^ channel model recorded with an ideal voltage-clamp (Figure 2 and Figure S2). At a fixed membrane potential the simulated current through deterministic Na^+^ channels is constant, whereas the equivalent stochastic simulation reveals large fluctuations in the Na^+^ channel current (Figure 2A). With sufficiently long periods of simulated channel activity, the mean amplitude of the stochastic current converges to the amplitude of the deterministic current (Figure 2B) and the estimated variance of the stochastic current converges to the value predicted from the number of channels and their single channel current amplitude (Figure 2C). In deterministic simulations positive voltage steps from a negative holding potential elicit smoothly varying inward currents that activate rapidly, inactivate and are followed by a resurgent component after repolarization to the negative holding potential (Figure S2). In corresponding stochastic simulations the current response contains step-like fluctuations and differs from trial to trial, with the average waveform over many trials converging on the equivalent deterministic waveform (Figure 2D). To determine whether the expected number of single channels and their single channel conductance could be retrieved from the simulated macroscopic currents, we carried out variance-mean analysis [36], [37]. Both parameters could be estimated from either the activating (Figure 2E) or the inactivating phase of the current (Figure 2F). Estimates of the number of single channels (Figure 2G) and their single channel conductance (Figure 2H) converged over many simulations onto their predicted values. These data demonstrate that our modified tau leap algorithm accurately simulates stochastic voltage-gated ion channel activity. This is further illustrated by the convergence towards zero of the error for the fit of the variance-mean function (Figure 2I). On the order of 10^4^ simulations were required for the fits to reliably converge to within 1% of the actual values, highlighting the importance of obtaining large numbers of repeated observations for estimating single channel properties using variance-mean analysis. Estimates obtained from 10^4^ or fewer simulations varied around the actual values depending on the number of simulations used (Figure 2G–I), suggesting an additional use of PSICS to quickly simulate the range of errors likely for estimates obtained from variance-mean analysis of macroscopic currents generated by channels with different gating schemes.

### Propagation of current and voltage in compartmental models containing stochastic ion channels

**Figure 3:**
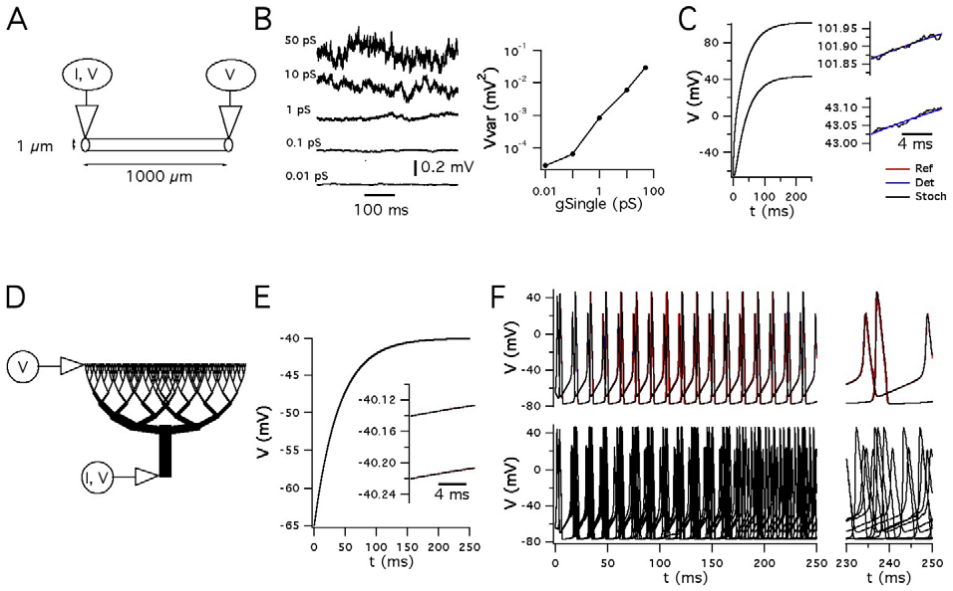
Simulation of membrane polarization and spike propagation in cable structures containing stochastic ion channels. (A) Simulated uniform cable with recording and current injection sites. (B) Example membrane potential traces (left) from simulations using leak Na^+^ and K^+^ channels with the indicated single channel conductances. The membrane potential variance is plotted as a function of the single channel conductance (right). (C) Voltage responses to injection of a current step at one end of the cable. Simulated stochastic (Stoch) and deterministic (Det) responses are plotted along with the analytical solution (Ref). Insets show the voltage as the current responses approach their steady-state values. In each case the traces overlap. As a result the Ref and Det traces are obscured by the Stoch trace. (D) Simulated branched cable structure with recording and current injection sites. (E) Voltage responses to injection of a current step at the base of the tree. Insets show the voltage as the current responses approach their steady-state values. Labels are as in (C). Ref and Det traces are obscured by the Stoch trace. (F) Action potentials generated when Hodgkin-Huxley channels are inserted into the cable in A. The top panel compares results of deterministic simulations using NEURON or PSICS, with stochastic PSICS simulations using a single channel conductance of 0.01 pS. The lower panel shows the output of several stochastic PSICS simulations using a single channel conductance of 20 pS. Membrane potentials in (E) and (F) are labeled as in (C), except that the blue trace is data from a simulation using NEURON.

Before comparing simulations of neurons with different morphologies, we first established the accuracy of simulation of current and voltage propagation using standard compartmental models for which there are analytical descriptions of the equivalent cable structures [38]. We examined the membrane potential of a simple cable containing stochastic leak Na^+^ and K^+^ channels (Figure 3A). While fluctuations in the membrane potential are very small when both leak channels have a very small single channel conductance (0.01 pS), when either single channel conductance is increased to physiological values (>1 pS), there is a substantial increase in the membrane noise (Figure 3B). Although of physiological relevance, this noise makes comparison with analytical results problematic and therefore for validation of stochastic simulations we used a single channel conductance of 0.01 pS. Membrane potential responses to injection of a current step at one end of the cable, simulated with PSICS using either stochastic or deterministic ion channels, were effectively identical to the analytical result (Figure 3C). Moreover, using a model of a branching dendrite (Figure 3D), stochastic or deterministic ion channel simulations with PSICS also accurately reproduce the predicted voltage change in response to current injection (Figure 3E). Thus, PSICS accurately simulates passive propagation of signals in compartmental models of cable structures, and when stochastic ion channels have very small single channel conductance the electrical behavior of a multi-compartment model is similar to models containing deterministic ion channels.

We next assessed simulation of excitable neurons. In a model of a cylinder containing active membrane conductances [38], simulations with PSICS that used deterministic ion channel models or stochastic implementations of channels with very small single channel conductance, produced essentially identical results to well established deterministic simulation software (Figure 3F). By contrast, when we increased the single channel conductance to more physiological values, we found that while the action potential waveform was similar, the stochastic ion channel gating introduced jitter into the timing of the action potentials, such that reproducible timing of spike firing was not maintained between trials (Figure 3F). We also compared simulation of an excitable model of a CA1 pyramidal cell shown in Figure 1, with published data obtained with the same model [39]. The initiation and back propagation of action potentials were reproduced by simulation of this model with PSICS using either deterministic or stochastic ion channels (data not shown).

### The consequences of stochastic gating depend on channel kinetics

**Figure 4:**
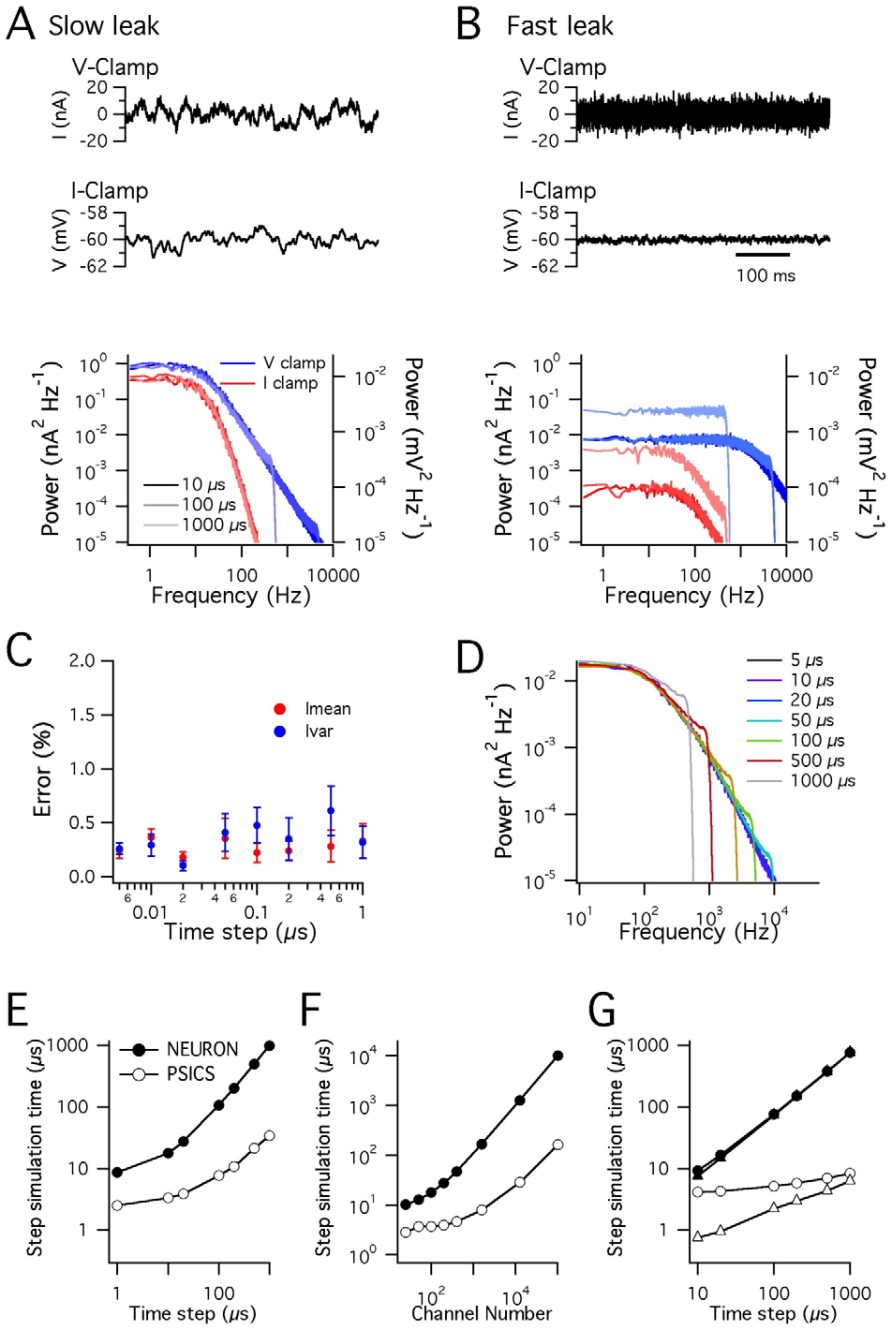
Relationships between channel kinetics, simulation configuration, accuracy and efficiency. (A–B) Examples of membrane currents (top), membrane potential (middle) and corresponding power spectra from 100 s of simulated activity (bottom), for models containing passive leak Na^+^ and K^+^ channels with slow (A) or fast (B) kinetics. The power spectra are shown for simulations with time steps of 10 µs (solid traces), 100 µs and 1000 µs (light traces). The voltage-clamp simulations are of a single isopotential compartment containing 201 Na^+^ and 1407 K^+^ leak channels. The current-clamp simulations are for a cable of length 1000 µm and radius 1 µm, containing 8050 channels distributed across 237 compartments. (C) The error in the mean and variance of a simulated current, mediated by 50 Na^+^ channels clamped at −20 mV for 100 s of simulated time, is plotted as a function of the simulation time step. (D) Power spectra for the currents in (C). Long time steps fail to simulate high frequency current fluctuations and introduce aliasing effects at low frequencies. (E–F) The computation time per simulation time step, required by NEURON (closed symbols) or PSICS (open symbols) for simulations as in (C–D), is plotted as a function of the duration of the simulation time step (E), or as a function of the number of simulated channels when the time step is 20 µs (F). (G) The computation time per simulation time step, required by NEURON or PSICS for simulations as in Figure 3F, is plotted as a function of the duration of the simulation time-step. Simulation times are for a cable divided into either 101 compartments (triangles) or 1001 compartments (circles).

While open probability and single channel conductance influence the amplitude of current fluctuations generated by stochastic ion channel gating, little attention has been given to the functional impact of channel kinetics or of interactions between channel properties and the membrane capacitance. The simplified models we used to evaluate PSICS also allowed us to begin to investigate these issues. To avoid non-linearities from voltage-dependent gating, we simulated single-compartment models that contain only passive leak Na^+^ and K^+^ channels. Each channel has one open and one closed state, with an open probability of 0.7, and the relative density of the channels was adjusted to produce a resting membrane potential of −60 mV. We compared a version of the model in which the forward and reverse rate constants for entering the open state were 0.07 ms^−1^ and 0.03 ms^−1^ (slow gating) with a version in which the corresponding rate constants were 7 ms^−1^ and 3 ms^−1^ (fast gating). The model containing the slow gating channels produced membrane currents in voltage-clamp, or membrane potentials in current-clamp, that fluctuated at frequencies below approximately 15 Hz (Figure 4A). By contrast, channels with faster gating kinetics produced current fluctuations with similar total power, but smaller amplitude at low frequencies (<∼15 Hz) and larger amplitude at higher frequencies (>∼15 Hz) (Figure 4B). In current-clamp simulations, the corresponding high-frequency membrane potential fluctuations were filtered by the membrane capacitance. As a result, the membrane potential fluctuations span a similar range of frequencies to the slow gating model, but have substantially smaller amplitude (Figure 4B). Thus, the functional impact of stochastic channel gating is determined by gating kinetics in conjunction with the membrane capacitance, as well as by open probability and single channel conductance.

To examine how the choice of a suitable simulation time step is constrained by these properties, we initially used the simple passive models described above. For the model containing slow leak channels, simulations with time-steps as large as 1 ms reproduced the dominant components of the power spectra of current and voltage fluctuations (Figure 4A). By contrast, for the model containing fast gating channels, simulation time-steps of 0.01 ms were required to satisfactorily simulate fluctuation of voltage-clamped currents, whereas time steps of duration up to 0.1 ms were sufficient to simulate membrane potential fluctuations in current-clamp conditions (Figure 4B). Thus, selection of the simulation time-step requires evaluation of the recording configuration, the power spectra of channel activity, the membrane time constant and the kinetics of the simulated channels.

### Efficient simulation of current and voltage propagation

Since simulation of complex neuronal morphologies can take considerable time, even using optimized computational algorithms, before simulating neuronal morphologies we first investigated strategies to minimize the time required for simulations without affecting accuracy of the results. We evaluated a stochastic implementation of the Hodgkin-Huxley Na^+^ channel model in a single compartment voltage-clamped at a fixed potential (Figure 4C–D). With simulation time-steps in the range of 1–1000 µs, currents simulated using PSICS had mean and variance that correspond well to the predicted values (Figure 4C). However, as the duration of the time-step is increased, the power spectra of the simulated currents reveal aliasing-like effects and failure to accurately simulate high frequency fluctuations (Figure 4D). Thus, as we expect from the properties of the tau leap algorithm, longer time steps will produce currents with the correct variance and mean amplitude, but will not accurately simulate high frequency components of current fluctuations.

To determine if improvements in simulation efficiency expected from use of the modified tau leap algorithm and an optimized computational core translate into practical reductions in simulation time, we compared the time required for simulations using PSICS to simulations run in the widely used NEURON simulation environment [40]. Stochastic ion channel gating can be simulated in NEURON using the next-transition algorithm which tracks the exact times at which each channel changes state [34], [35]. For simulations of voltage-clamped currents using short time-steps and relatively few ion channels, the simulation time with PSICS was approximately three-fold faster than using NEURON (Figure 4E–F). This difference increased to a more than 10 fold reduction in simulation time when PSICS is used for simulations with larger time steps and more ion channels. We next evaluated performance using the spiking single cable model also used for the simulations in Figure 3F. This model contains several types of ion channel distributed across multiple compartments and has a rapidly fluctuating membrane potential. The simulation time per unit biological time was constant for simulations run with NEURON and was independent of the compartment size. By contrast, the times for simulations run with PSICS were faster at all time steps. This difference was >100 fold with relatively large numbers of channels per compartment and long time-steps (Figure 4G). For simulation parameters likely to be appropriate for many detailed neuronal models we estimate that PSICS obtains approximately an order of magnitude or greater reduction in simulation time. Together, these data establish that the new tools we have developed enable accurate and efficient stochastic simulations of neurons with complex morphologies, with performance that is superior to other general-purpose software.

### Morphology and kinetics interact to determine the influence of stochastic gating on membrane potential

**Figure 5 pcbi-1000886-g005:**
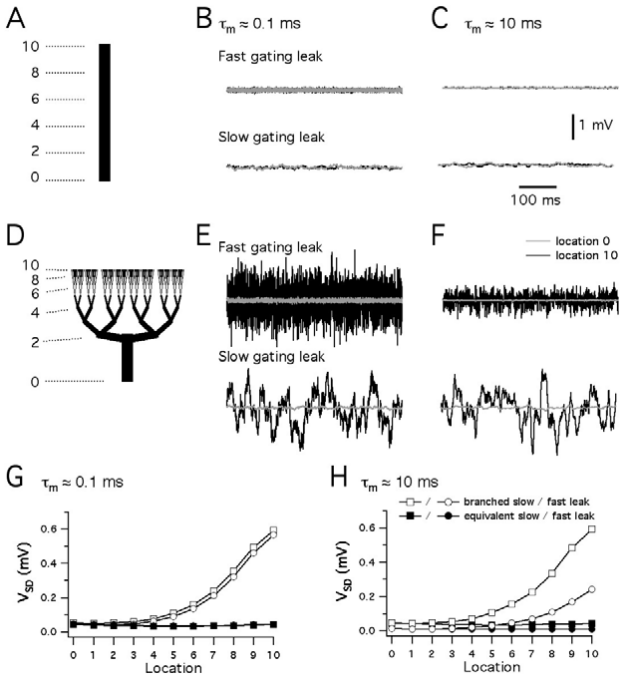
Dendrite morphology determines the influence of stochastic channel opening on membrane potential. (A–F) Recordings of resting membrane potential at proximal (grey traces) and distal (black traces) locations on a multi-compartmental model of a cylinder of length 320 µm and diameter 16 µm (A–C) or a hypothetical branched dendrite (D–F). The cylinder in (A–C) is electrically ‘equivalent’ to the dendrite in (D–F), which has a branching organization that follows Rall's 3/2 power law. The distal recordings are from location ‘10’ and the proximal recordings are from location ‘0’. In each panel the membrane potential when the leak channels have fast kinetics (upper traces) is compared to the membrane potential when the leak channels have slower kinetics (lower traces). Membrane potential when the models have a membrane time constant on the order of 0.1 ms (B,E) is compared to models with a membrane time constant on the order of 10 ms (C,F). The scale bars apply to all traces. (G–H) The standard deviation of the resting membrane potential of the models in (A–F) is plotted as a function of recording location. Each point is the average of data from 5 simulations of 1s of neuronal activity. The same data were used for statistical analysis (ANOVA). Black and grey symbols correspond to distal and proximal recording locations as in (A–F) above.

Does morphology influence the functional consequences of stochastic ion channel gating? To address this possibility, we first compared the membrane potential noise resulting from stochastic ion channel gating in a hypothetical dendritic tree that obeys Rall's 2/3 power law, with membrane potential noise resulting from stochastic ion channel gating in the corresponding equivalent cable structure [41] (Figure 5). In both structures spontaneous opening and closing of fast leak K^+^ and Na^+^ channels causes noisy fluctuations in the membrane potential. These fluctuations increase in amplitude by more than ten fold between the proximal and the distal ends of the branched dendrite model (Figure 5D–E)(p<<1e-9), but have relatively small amplitude throughout the equivalent cylinder (Figure 5A–B)(p = 0.35). Similar differences in amplitude and location-dependence were present for models that instead contained the slow gating leak channels, but were otherwise identical (Figure 5B, E).

Under what conditions do channel kinetics determine the impact of stochastic ion channel gating? Whereas our earlier simulations indicated an important role for channel kinetics (Figure 4A–B), in our initial simulations of the hypothetical dendritic tree and equivalent cable the kinetics of the leak current did not affect the amplitude of the membrane potential fluctuations (p = 0.63)(Figure 5G). However, since the branching tree and the equivalent cable have a membrane time constant on the order of 0.1 ms, whereas many central neurons have membrane time constants on the order of 10 ms, we re-evaluated these models after increasing the membrane capacitance to bring the time constant into this range (Figure 5C, F). In this case, the amplitude of the membrane potential fluctuations was also dependent on location in the branched dendrite (p<<1e-9)(Figure 5F), but not in the equivalent cable (p = 0.474)(Figure 5C). Moreover, in contrast to the models with the fast membrane time constant, for models with a more physiological membrane time constant the kinetics of the leak current profoundly influenced the amplitude of membrane potential fluctuations (Figure 5F). In models containing the fast leak channels the amplitude of membrane potential fluctuations was reduced (p<<1e-9), but in models containing the slow leak channels their amplitude was similar (p = 0.54)(cf. Figure 5G,H). Thus, the cellular effects of stochastic ion channel gating depend on morphology, while the specific effects of morphology depend on the kinetics of the ion channels found in the membrane. Since the consequences of stochastic ion channel gating are sensitive to their specific cellular context, establishing the impact of stochastic gating in particular central neurons will require simulations account for details of their morphology.

### Functional effects of stochastic gating depend on neuronal morphology

**Figure 6 pcbi-1000886-g006:**
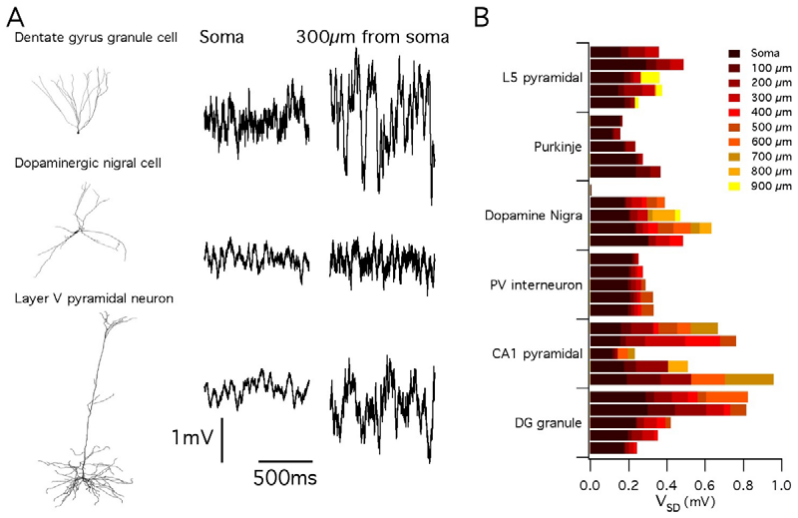
Consequences of stochastic ion channel gating differ between morphologically distinct neuronal cell types. (A) Examples of membrane potential (right) and corresponding morphology (left) from a simulated dentate gyrus granule cell (top), dopaminergic nigral cell (middle) and cortical layer V pyramidal cell (bottom). All models contained identical ion channel distributions. (B) Average membrane potential standard deviation for model neurons of each morphological type plotted as a function of increasing distance along the dendrite from the soma. The membrane potential standard deviation at a particular location corresponds to the right most end of the bar indicated by the corresponding color. The standard deviation increases with distance from the soma.

Do realistic neuronal morphologies influence the functional impact of stochastic ion channel gating? While the simulations described above suggest this may be the case, they also suggest that the consequences of stochastic gating depend on the specific details of neuronal morphology and ion channel kinetics. To address this question directly, we therefore reasoned that if neuronal morphology is an important determinant of the impact of stochastic ion channel gating, then simulations using identical rules to introduce identical stochastic ion channels into neurons with distinct dendritic morphologies, should predict differences between neurons (Figure 6). We simulated resting membrane potential activity in 29 reconstructed neurons downloaded from neuroMorpho.org [42]. The neuronal models spanned 6 distinct morphological classes: neocortical layer V pyramidal neurons (n = 5), cerebellar Purkinje neurons (n = 5), dopaminergic substantia nigra neurons (n = 4), parvalbumin-positive interneurons (n = 5), hippocampal CA1 pyramidal neurons (n = 5) and hippocampal dentate gyrus granule cells (n = 5). Fluctuations in the membrane potential were apparent in all neurons simulated using stochastically gating ion channels (Figure 6A). However, the amplitude of these fluctuations varied significantly both between neurons of the same morphological class (p<0.01 for all classes), between neurons of different morphological classes (p<<1e-9 (Figure 6B), and as a function of dendritic location within neurons (p<<1e-9). For example, pyramidal neurons from the neocortex demonstrate relatively small amplitude membrane potential fluctuations (Figure 6). This is consistent with a previous modeling study of stochastic ion channel activity in a single layer V pyramidal neuron [43]. By contrast, membrane potential fluctuations could be substantially larger in hippocampal dentate gyrus granule cells (Figure 6). Thus, the impact of stochastic gating of dendritic ion channels on neuronal electrical properties is determined by neuronal morphology and can vary according to dendritic location.

**Figure 7 pcbi-1000886-g007:**
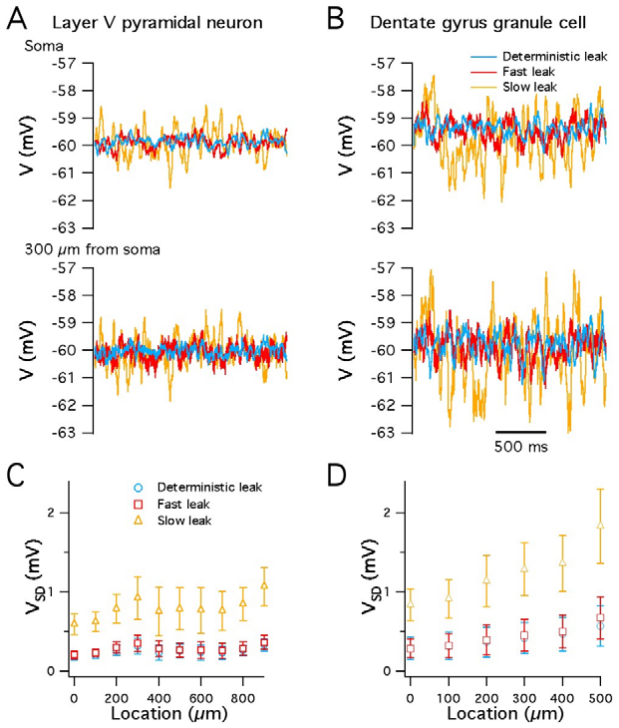
Channel kinetics determine the functional impact of stochastic gating. (A–B) Simulated membrane potential of a model layer V pyramidal neuron (A) and a dentate gyrus granule cell (B) containing either a deterministic leak conductance, fast gating stochastic leak channels or slow gating stochastic leak channels. (C–D) Mean variance of membrane potential fluctuations, recorded from simulated layer V pyramidal neurons (C) and simulated dentate gyrus granule cells (D), plotted as a function of distance from the cell body.

As the impact of stochastic gating in the abstract models described above depended on channel kinetics (Figure 4A–B and Figure 5), we asked if this is also the case in the models based on reconstructed neurons. We focused on models of cortical layer V pyramidal neurons and on models of granule cells from the dentate gyrus of the hippocampus. When the fast gating leak channels used for the simulations in Figure 6 were replaced with an equivalent deterministic conductance, we found almost no difference in the amplitude of membrane potential fluctuations recorded from somatic or dendritic locations (DG neurons average 1.11 fold difference, p = 0.02; Layer V neurons, average 1.14 fold difference, p = 1.5e-6) (Figure 7). Thus, in the configuration used for simulations in Figure 6, the membrane potential fluctuations are primarily driven by stochastic gating of voltage-gated ion channels, but not by the leak channels. By contrast, when we replaced the fast gating leak channels with otherwise identical slow gating leak channels, the membrane potential fluctuations were approximately three-fold larger than fluctuations recorded from models containing deterministic or fast-gating stochastic leak channels (DG neurons average 3.13 fold difference, p<<1e-9; Layer V neurons, average 3.08 fold difference, p<<1e-9) (Figure 7). Thus, slow gating leak channels can increase the amplitude of spontaneous membrane potential fluctuations. This suggests a novel mechanism for modulation of neuronal activity, whereby modulation of channel gating, without affecting open probability or single channel conductance, could profoundly influence fluctuations in a neuron's somatic or dendritic membrane potential.

### Stochastic gating of dendritic and axonal ion channels modifies synaptically driven spike output from a detailed model CA1 pyramidal neuron

**Figure 8 pcbi-1000886-g008:**
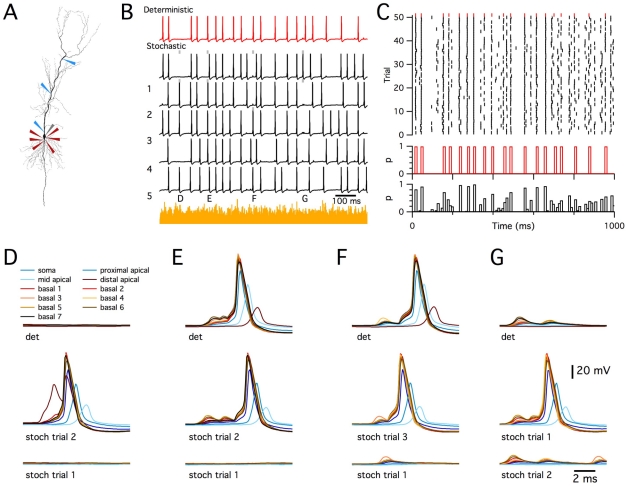
Stochastic ion channels modify synaptically driven spike output from a CA1 pyramidal neuron. (A) Morphology of the simulated CA1 pyrmidal neurons (described in [44]), illustrating positions of recording electrodes placed on the soma (grey), apical (blue) and basal (red) dendrites. (B) Examples of membrane potential responses of the deterministic (red trace) and stochastic (black traces) versions of the model to distributed synaptic input. The summed synaptic current is shown in yellow. Letters and grey bars indicate times of action potentials highlighted in subsequent panels. (C) Spike rasters (top) for responses of the determinisitc model (red) and for 50 consecutive trials of the stochastic model (black) to the synaptic input pattern used in (B). Plotted below is the probability of somatic spike firing in 10 ms duration bins for the deterministic (red) and stochastic (black) versions of the model. (D–G) Examples of determinisitc responses (top row) and representative stochastic respones (lower two rows), illustrating “extra” somatic action potentials triggered by an “extra” actively propagating dendritic spike (D), “dropped” somatic action potentials resulting from failed dendritic depolarization (E–F), and an “extra” somatic action potental resulting from additional dendritic depolarizing potentials.

The previous simulations establish that in principle stochastic gating of intrinsic ion channels might be important for neuronal function, but the impact of stochastic ion channel gating on neuronal responses to physiological patterns of synaptic input is not known. We therefore asked if stochastic gating of post-synaptic ion channels in dendritic neurons influence the transformation of synaptic input into spike output obtained with realistic neuronal morphologies and ion channel properties? In the models described so far, ion channel distributions were chosen to facilitate comparisons between morphologies. To address more realistic ion channel distributions we adopt a model of a CA1 pyramidal neuron that has previously been shown to account well for somatic and dendritically initiated action potentials [44] (Figure 8A). To further increase the approximation of the model to a real CA1 pyramidal neuron we introduced HCN channels with distribution following previous experimental descriptions [45]–[47]. We then examined responses of the model to ongoing activation of 1502 synaptic inputs distributed throughout the basal and apical dendrites, each activated independently according to a Poisson process with an average frequency of 5.5 Hz. We focus here on results of simulations in which the neuron was driven by synaptic input to fire at frequencies of approximately 20 Hz, which is towards the upper end of firing frequency of active CA1 neuron *in vivo* [48]. Similar results are obtained for synaptic input that drives firing at lower frequencies and when synaptic input is distributed so that spikes are triggered primarily by depolarization of the distal apical dendrites (not shown). We consider here only asynchronous and distributed synaptic input, which is likely to correspond to activity during the theta state in awake animals [49]. As in experimental studies [49], [50], forward propagating apical dendritic spikes were only evoked in the model using highly coincident and spatially localized stimuli, but were not observed in response to the patterns of input that we investigate here.

Compared to the deterministic model, the stochastic version generated “extra” spikes at times when the equivalent deterministic neuron was silent and “dropped” spikes at times when the equivalent deterministic neuron fired action potentials (Figure 8B). Importantly, the “extra” spikes observed during simulation of the stochastic model occurred at similar time-points from trial to trial (Figure 8C and Figure S3). Thus, stochastic channels allow probabilistic detection of features in the stimulus waveform that would not produce responses in a deterministic neuron. Likewise, not all spikes observed in the deterministic simulation were “dropped” in the stochastic simulation, but rather “dropped” spikes were more likely at particular time points (Figure 8C and Figure S3). Comparison of spike times from repeated trials demonstrated that stochastic ion channel gating also introduced considerable jitter into the timing of action potentials. Therefore, whereas deterministic neurons encode information using a fixed response to particular patterns of synaptic input, these results suggest that stochastic gating of intrinsic ion channels enables pyramidal neurons to generate probabilistic responses. Thus, while for both stochastic and deterministic neurons certain combinations of synaptic input evoke spikes with high reliability and other combinations fail to elicit spikes, in stochastic neurons some patterns of synaptic input have an intermediate probability of evoking spikes, which is observed as trial-to-trial variability (Figure 8C). While this intermediate probability might not be decoded in a single trial from a single neuron, if each trial is instead considered as the response of a different neuron within a large population, then the probabilistic responses could quite easily be decoded from the population activity (Figure 8C).

To evaluate the mechanism for probabilistic initiation of action potentials, we recorded membrane potential from the soma and from proximal parts of each primary dendrite. While some somatic action potentials were preceded by dendritic depolarizations that resemble fully propagating dendritic spikes (Figure 8D), most were preceded by smaller amplitude dendritic depolarizations (Figure 8E–G). The all-or-nothing nature of these smaller events suggests that they reflect dendritic action potentials that propagate passively to the soma (Figure 8E–G and Figure S3). This is consistent with experimental recordings from basal dendrites of cortical pyramidal neurons [51]. In the stochastic model “extra” somatic spikes could result from additional actively propagating dendritic spikes (Figure 8D) or additional smaller all-or-nothing dendritic depolarizations of sufficient amplitude to elicit somatic action potentials (Figure 8G), while “dropped” somatic spikes resulted from failures to initiate all-or-nothing dendritic depolarizations (Figure 8E–F). These observations point to the importance of local dendritic signaling for the functional consequences of stochastic ion channel gating in pyramidal neurons and suggest that synaptic initiation and active propagation of dendritic spikes may be particularly sensitive to stochastic membrane potential fluctuations.

**Figure 9:**
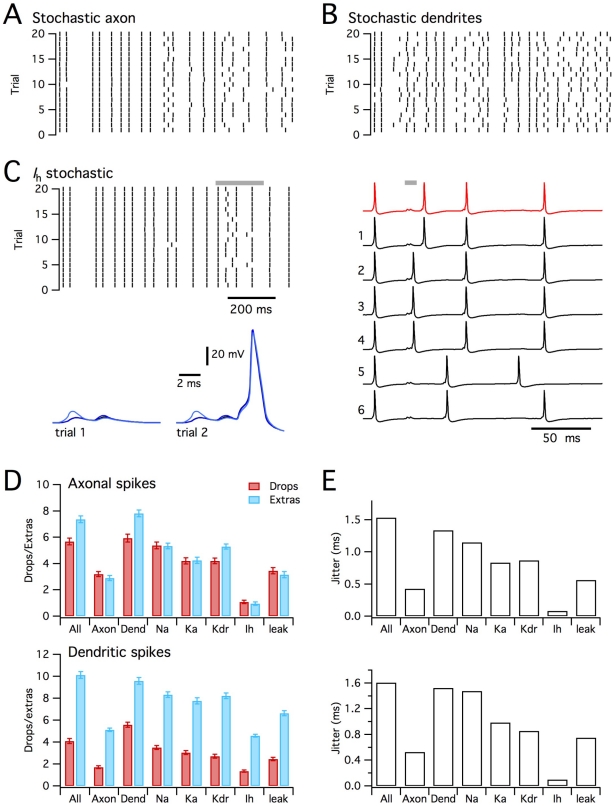
Differential impact of distinct ion channel types and locations. (A–B) Rasters for first 20 trials of responses of the CA1 pyramidal neuron simulated as in Figure 8, but with stochastic axonal and deterministic dendritic ion channels (A) or stochastic denritic and deterministic axonal ion channels (B). (C) as for (A–B), but only channels mediating *I*_h_ are stochastic. Shown are rasters (left), examples of membrane potential responses of the fully deterministic model (red) and first six sweeps recorded from the stochastic model (black), and examples of membrane potential waveforms corresponding to an “extra” action potential triggered by an additional dendritic depolarization. (D) Number of “dropped” and “extra” axonal spikes (top) and dendritic spikes (bottom) during 1s of simulated time for each experimental condition tested. Because of their all or nothing nature, large dendritic depolariations are classified as spikes. ANOVA indicated a significant (p<<1e-9) effect of model configuration on “dropped” and “extra” axonal and dendritic spikes. Key post-hoc comparisons are referred to in the main text. (E) Jitter in the timing of axonal (top) and dendritic (bottom) action potentials for each experimental condition.

To evaluate the relative roles of stochastic axonal compared with stochastic dendritic ion channels we implemented versions of the model in which one population of ion channels was deterministic and the other stochastic. Both axonal and dendritic stochastic channels caused “dropped” and “extra” dendritic spikes (Figure 9A–B, D). When only axonal channels were stochastic, the number of “dropped” and “extra” somatic spikes (p<1e-6 in both cases) and dendritic spikes model (p<1e-6 in both cases) were less than in the fully stochastic model (Figure 9A). In contrast, when only the dendritic channels gated stochastically, we found that the number of “dropped” and “extra” somatic spikes was not significantly different compared to the fully stochastic model (p = 0.99 and p = 0.85 respectively), while the number of “extra” (p<1e-6), but not the number of “dropped” (p = 0.81) dendritic spikes differed from the fully stochastic model (Figure 9B). The number of “dropped” and “extra” spikes was much smaller in the stochastic axon model, compared with the stochastic dendrite model (p<1e-6 in all cases). Stochastic gating of axonal channels also caused very little additional jitter in the timing of action potentials, whereas stochastically gating dendritic ion channels could account for almost all of the spike jitter (Figure 9E). These data suggest that while stochastic gating of axonal channels can modify spike patterns, stochastic dendritic channels account for most of the impact of stochastic gating on synaptically driven spike output. This is consistent with the substantial effects of stochastic gating on initiation of dendritic spikes (Figure 8D–G and Figure S3).

Since the model CA1 pyramidal neuron contains several types of ion channel that differ in their kinetics, voltage-dependence and single channel conductance [44], we asked if any particular channel type mediates the consequences of stochastic gating? We compared versions of the model in which only one type of ion channel was simulated stochastically and the others were simulated deterministically. These simulations demonstrated that stochastic gating of any single type of ion channel is insufficient to fully account for all of the “dropped” or “extra” spikes observed in the fully stochastic model (Figure 9D–E). The greatest impact on spike output came from stochastically gating voltage-dependent Na^+^ channels, followed by A-type and delayed rectifier potassium channels (Figure 9D–E and Figure S4). Thus, the number of “dropped” somatic and dendritic spikes did not differ between the model in which only Na^+^ channels gated stochastically compared with the fully stochastic model (p = 0.98 and 0.3), whereas there were fewer “extra” somatic and dendritic spikes (p<1e-4 in both cases). Models in which only one of the other ion channel types gated stochastically differed significantly from the fully stochastic model in all measures of “extra” and “dropped” spikes (p<1e-3). Nevertheless, models containing stochastic gating voltage-dependent K^+^ channels generated considerably more than 50% of the number of “extra” and “dropped” spikes observed in the fully stochastic model. Interestingly, stochastic gating of *I*_h_ channels alone had very little impact on axonal spikes or spike jitter, but nevertheless increased the number of “extra” dendritic spikes. The relative lack of effect of *I*_h_ can be explained by the small single channel conductance, while the primary effect on additional dendritic spikes may reflect slow gating kinetics and dendritic localization of these channels (Figure 9C). Together, these data suggest that in a well-validated, realistic model of a CA1 pyramidal neuron experiencing distributed synaptic input sufficient to drive action potentials at physiologically relevant rates, stochastic gating of dendritic ion channels substantially modifies spike output. While no single ion channel is sufficient to fully account for modified spike output, stochastic gating of dendritic voltage-gated Na^+^ and K^+^ channels may be particularly important.

## Discussion

To address the functional consequences of stochastic gating of neuronal ion channels we developed and validated new, efficient and general-purpose tools for numerical simulation of cells with complex morphologies. Using these tools we have made several new findings. First, we show that the functional impact of stochastic ion channel gating depends on neuronal morphology and as a result differs between neuronal cell types. Second, we show that depending on a neuron's morphology, ion channel kinetics influence the functional consequences of stochastic ion channel gating. These results suggest that detailed and well-constrained simulations will be important for accurate prediction of the specific functional consequences of stochastic gating in particular cell types. Third, we show that in a realistic model CA1 neuron, stochastic gating of non-synaptic ion channels modifies the timing of synaptically driven somatic and dendritic action potentials, and causes substantial numbers of “extra” and “dropped” somatic and dendritic spikes compared to equivalent deterministic neurons. These results suggests a new perspective on dendritic integration of synaptic inputs, whereby stochastic gating of intrinsic ion channels enables populations of neurons to compute using probabilistic rather than fixed spike codes.

### Functional consequences of stochastic ion channel gating depend on neuronal morphology and channel properties

Gating of single ion channels is one of the better-understood stochastic processes in biology [24], [30], [52]. Nevertheless, the functional consequences of discrete transitions between open and closed states of ion channels found in the membranes of morphologically complex neurons are not well understood and for technical reasons have received relatively little attention. The reductions in computation time obtained with PSICS enable this issue to be addressed systematically for the first time using detailed simulations of large numbers of reconstructed neurons (Figures 4–9). Modification by stochastic ion channel gating of the pattern and timing of spikes generated in response to synaptic input to a previously well validated model CA1 pyramidal neuron (Figures 8 and 9), suggests that stochastic ion channel gating substantially influences synaptic integration by dendritic neurons. However, our simulations also suggest reasons for caution in extrapolating between cell types as the consequences of stochastic gating depended on neuronal morphology (Figures 5 and 6). Thus, the impact of stochastic opening and closing of ion channels varies as a function of sub-cellular location within a neuron and may differ both between neuronal cell types and across neurons of the same cell type. Moreover, our finding that ion channels with identical open probability, but distinct gating kinetics produce different membrane potential fluctuations (Figure 7), while suggesting a previously unexplored mechanism for control of neuronal activity, also indicates that single-channel recordings of ion channels in dendrites and axons (e.g. [53]–[55]) will be important to constrain stochastic models of excitable neurons. Given debates over the accuracy of aspects of reconstructed neuronal morphologies [56], [57], our comparison of neuronal cell types should be considered as a proof of principle rather than a definitive description of a particular neurons activity. Since experimental tests that replace a neurons stochastic with equivalent deterministically gating dendritic ion channels are not currently possible, accumulation of accurate morphological and biophysical data will be particularly important for further investigator of the roles of stochastic gating in particular cell types.

Irrespective of the details of any particular model, our results suggest that neuronal morphology and ion channel properties interact to determine the functional consequences of ion channel gating. Comparison of model neurons with different morphology, but containing identical ion channels, indicates that dendritic morphology plays a key role in determining the functional consequences of stochastic ion channel gating (Figures 6–7). Diversity between neurons *in vivo* in their expression of particular ion channels [58], could accentuate or attenuate distinctions between neurons predicted on the basis of their morphology. Simulations of the detailed CA1 pyramidal neuron model in which only one ion channel type was implemented stochastically suggest several further insights into the roles of particular types of ion channel. First, stochastic gating of any single ion channel type was insufficient to fully account for probabilistic behavior of the fully stochastic neuron. Second, quantitative differences between the probabilistic behavior of the fully deterministic and stochastic neurons were considerably less than the sum of the differences between the fully deterministic neuron and each version of the model in which only one ion channel type gated stochastically. This suggests considerable redundancy in the functional consequences of gating by any particular type of ion channel. Third, stochastic gating of only a single ion channel type, for example voltage-gated Na^+^ channels in Figure 9, can nevertheless substantially modify spike output. The latter two conclusions suggest that the results of our simulations will be robust to different assumptions about single channel properties of particular ion channels and at worst may under-estimate the influence of stochastic ion channel gating (see Methods). Fourth, the impact of stochastic gating differs between ion channels types. For example, compare the model CA1 pyramidal neuron in which only *I*_h_ channels gate stochastically, with equivalent models in which other ion channels gate stochastically (Figure 9). The relatively small impact of *I*_h_ is perhaps not surprising given the relatively small underlying single channel conductance, which is estimated to be on the order of 1 pS [13]. Given that *I*_h_ is a major contribution to the resting dendritic membrane conductance of pyramidal neurons [13], [46], it might at first appear surprising that stochastic gating of other ion channel types can have such large effects. However, we have previously shown that as HCN channels are deactivated by depolarization, at potentials closer to spike threshold their impact is minimal compared to stochastic gating of other types of voltage-dependent ion channel [11], [59]. The substantial influence on synaptically driven spike output of stochastic gating of voltage-gated Na+ and K+ channels is consistent with this explanation (Figure 9).

### Simulation of stochastic ion channels in cells with complex morphology

Since it is rarely possible to reduce electrical activity within morphologically complex excitable neurons to tractable analytical models, mechanistic simulation of axons and dendrites relies on well-constrained compartmental models. Compartmental simulations necessarily involve trade-offs between accuracy and simulation time. This is a particularly important problem for simulations that aim to account for the stochastic transitions between the states of each individual ion channel in a realistic model neuron. To enable efficient and accurate simulation, we adopted an algorithm that generates a correct distribution of ion channel states at each simulation time point, while sacrificing explicit representation of ion channel states during the interval between time points. Relatively short time steps are required for accurate simulation of voltage-clamped conductances with rapid kinetics (Figure 4). In contrast, during simulation of membrane voltage recorded in the current-clamp configuration, high frequency current fluctuations are filtered by the membrane capacitance and therefore have little impact on neuronal activity. Therefore, high frequency component of the conductance fluctuations do not have to be explicitly simulated (Figure 4A–B) and so simulations using the modified tau leap algorithm can take advantage of longer time steps.

Our new approach has several advantages over previous methods for simulation of stochastic ion channel gating. While approaches that add noise terms to the equations used to calculate the membrane currents are computationally efficient [14], [60], the noise term is at best only indirectly related to the biophysical properties of the simulated channels. Thus, these methods may be of use for efficiently simulating some of the phenomenological consequences of stochastic channel gating, but are of less utility for addressing the relationship between properties of single ion channels and macroscopic neuronal activity [22]. One approach to account for single channel properties is to explicitly track the probability that each channel makes a transition between states during each time step [11], [23]. However, this method is accurate only if sufficiently small time steps are used [22] and is computationally very expensive. An alternative method is to explicitly track the exact time of transitions of channels between states, while counting only the number of channels in each particular state [34], [35]. This demands less computation time than explicitly tracking each channel, but nevertheless requires generation of random numbers between time steps and therefore becomes inefficient for longer time steps or large numbers of channels. In contrast, PSICS uses a simulation algorithm that accurately simulates the distribution of ion channel states at each time step without having to track the exact time of each transition, while also counting only the number of ion channels in each state without having to track the states of individual channels. We show that this can lead to an order of magnitude or greater improvement in simulation time without loss of accuracy (Figure 4). For all approaches, including those introduced here, parallel computing produces a further linear reduction in computation time with additional processors simply by enabling the multiple simulations required for statistical evaluation of models to be carried out simultaneously.

### Probabilistic neurons and further functional consequences of stochastic ion channel gating

By implementing a previously well-validated model of a CA1 pyramidal neuron using stochastically gating ion channels, our simulation results provide evidence that synaptic integration by dendritic neurons is probabilistic. While the instantaneous output of a single neuron functioning in this way is relatively unreliable, instantaneous representations distributed across a population of stochastic neurons could be read out by summation of their outputs. The impact of such probabilistic integration on information processing and computation by populations of pyramidal neurons remains to be determined. For CA1 pyramidal neurons in the hippocampus, one possibility is that this probabilistic integration is important for encoding of location by the timing of action potentials relative to ongoing network rhythms [61]. Indeed, our results are consistent with relatively unreliable encoding of location by the timing of individual action potentials, but suggest that coding mechanism might be considerably more reliable when the activity of large ensembles of neurons is considered.

Challenges for future experimental and theoretical studies include determining the conditions, additional cell types and sub-cellular locations in which stochastic gating of ion channels affects spike output, and to establish the consequences for computations carried out by neural circuits. At some sub-cellular locations noise introduced by stochastic gating of single ion channels might impose physical constraints on the computational properties of neurons [12] and may limit the efficiency of neural coding [62]. Alternatively, neuronal noise sources may promote detection of signals [63]–[66], enable multiplication of synaptic responses [67], or control the pattern of action potential firing [11]. The tools we introduce here will enable these and other possibilities to be addressed systematically. In addition to exploring physiological mechanisms, systematic simulations may be important for understanding the functional consequences of changes in morphology or ion channel localization that accompany nervous system disorders. For example, changes in dendritic morphology are reported in several forms of mental retardation [68], but the functional implications of interactions between stochastic gating of dendritic ion channels and disease or behaviorally related changes in dendrite morphology are yet to be addressed.

## Methods

All calculations were performed with PSICS (www.psics.org) unless indicated otherwise. Both the PSICS shell and ICING are written in Java and can run on Windows, OS X and Linux operating systems. To minimize the time required to run simulations, the default version of the PSICS core is written in Fortran and has been compiled separately for each operating system. A slower version of the core that runs in Java is also available. The core of PSICS performs equivalent deterministic or stochastic simulations of all models. In PSICS ion channels are treated as distinct entities rather than as a conductance density, and channel gating can be simulated either stochastically or in the deterministic limit. Most of the methods involved in such calculations are well documented elsewhere [9], [69]. Here we present the two novel aspects of the method: the way ion channels are positioned and mapped onto a discretization of the structure; and the approximations used to generate realizations of the stochastic behaviour of the system much more rapidly than is possible with previous stochastic methods. Further details of PSICS development are given in the Supplemental Text.

### Ion channel positioning

Channel positions are allocated according to user-specified probability densities over the structure such that each channel has a position in three dimensions. The input morphology is sub-sampled at 1 µm for computing local number densities. Axial positions for channels are generated either by sampling a Poisson distribution for the distance to the next channel, or by taking uniform increments to give the desired average density. The angles at which channels occur around a section are allocated randomly from a uniform distribution. At present, these angles only affect the visualization since the structure is later discretized into elements with end faces perpendicular to the axis. The seeds used for the random number generator are stored with the model so that exact allocations can be reproduced. So that allocations are portable across platforms, the generator used is a Mersenne Twister [70], which is included as part of PSICS rather than relying on a system library.

For computing the propagation of membrane potential changes, the structure is compartmentalized into elements such that all elements have approximately the same value of: 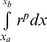


where 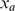 and 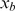 are the positions of the end points of the compartment along the structure, *r* is the local radius and 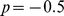 was used throughout this study. This is a purely geometrical property that provides a discretization that balances conductance between compartments against charging rates for membrane capacitance and is independent of the membrane time constant. PSICS allows post-hoc adjustment of the discretization in view of the conductances arising from allocated channels, but the facility was not used for the present study. In general, the resulting elements are neither straight nor of uniform radius so conductances between compartments are also computed as integrals along the structure.

### Realization of stochastic ion channel behavior

Ion channels are represented by kinetic schemes. Each scheme has one or more complexes, and each complex is an inter-connected set of states with expressions for the transition rates between them. Models using the Hodgkin-Huxley gating particle description are mapped to the corresponding scheme where each gate corresponds to a two-state complex [24]. For deterministic calculations, multi-complex schemes are used directly. For stochastic calculations, multi-complex schemes are mapped to the equivalent single-complex scheme by “multiplying-out” [24], [71]. For a scheme with *n* states, the probabilities of the channel being in each of the 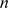 states can be expressed by a probability density vector 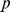 of length 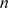, where 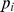 is the probability of being in state 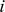. A channel in state 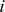 can only transition into one of the states directly connected to that state. If the classical transition rate between state 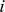 and state 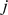 is 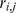 then when 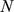 channels are in state 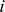, the number of transitions per unit time between from state 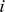 to state 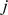 would be 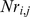 for large 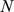 (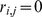 if there is no direct connection from states 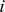 to state 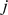). In this case, the single channel probabilities obey a similar relation giving the rate of change of 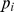 as the difference between the total fluxes into and out of that state:
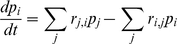



By gathering up the terms, this can be written in matrix form as a master equation [72]:
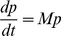
where,
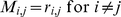
and
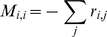



In general, 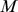 will depend on the membrane potential 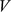, and could incorporate other dependencies such as calcium or other second messengers. Note that, as with the transition rates themselves, 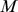 only has non-zero elements along the diagonal and where direct transitions are possible in the channel scheme. Using 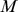 directly to update state occupancy probabilities and sample the resulting distribution to generate a stochastic simulation of the channel corresponds to the explicit method (e.g. [11]). If it is not necessary to follow every transition, then the simulation can be made more efficient by integrating the master equation over a time interval 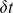 yielding:
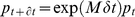



As this integral is exact for constant 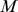, the state occupancy probabilities can be computed for any future time. In practice 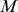 is often voltage dependent, so 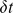 should be kept small enough that there are no significant changes in 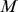 over a single time step.

In calculations, significant efficiency improvements over explicit methods can be achieved by using the fixed step transition matrix, 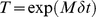, which allows channel to move through many briefly occupied states in a single calculation step without having to follow the dynamics of each transition. Unlike 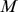, 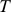 is typically non-zero everywhere, since there is a non-zero probability of the channel making the multiple transitions necessary to get from one state to any other state within the time step. For a deterministic calculation, the state occupancy probability vector 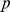 is replaced by an occupancy density vector, 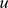, while the rest of the derivation remains the same, so the update step for 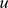 is:
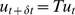



For a stochastic calculation, the element 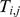 is the probability that a channel currently in state 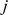 will be in state 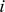 at the end of the time-step. The update step for a single channel involves generating a uniform random number, r, between 0 and 1 and then comparing it to the elements of the 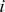
'th column of 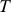, where 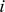 is the current state of the channel. The selected state, 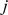, is such that:
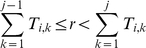



That is, the elements 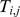 act as the widths of bins and the random number is used to pick one of the destination bins according to their relative sizes.

For small populations, the update step is applied to each channel individually. For larger populations, significant computation time can be saved by updating only the channels that have a non-negligible probability of changing state during the step and rescaling the bins accordingly. Thus, if the total number of channels in a given state is 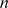 and the probability of any channel leaving the state in the next time step is 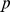 then the number of channels 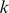 leaving the state in one step has a binomial distribution:
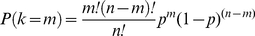
and the probability that 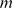 or more channels will leave is:
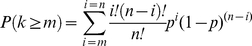



For a statistically reliable result, we require a bound on the number of missed events, here set at 2 in 10^8^. This requires 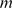 such that:
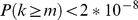



In general, however, finding the smallest 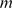 that meets this condition is a computationally costly process and, since it needs to be done for each state of each channel population at each time step, these costs could wipe out any benefits of not advancing the channels individually. To avoid this problem, the following fitting formulae are used to produce a safe estimate of 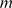 in terms of the probability 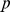 of leaving the current state, and the number of channels, 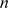, in that state:
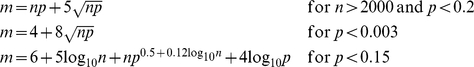



The first applicable formula is used. If none of the conditions is met, all the channels are advanced individually. These formulae were arrived at by a combination of experimentation and numerical optimization to find expressions that approximate the form of the exactly computed values of 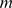 as a function of 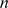 and 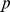 over a logarithmically sampled grid. They ensure that the chance of more channels making a transition out of the current state than are considered at one step is less than 2 in 10^8^.

For ion channels the modified tau leap method removes the major cost of the next transition method [34], [35], which lies in re-computing next transition times whenever the membrane potential changes significantly, even when no transition has occurred. In the tau-leap method, a different membrane potential simply requires the use of different transition probabilities for the next step. Since all the required tables can be pre-computed, then if the steps are small enough that no significant potential change occurs within a step (a condition that is easily met), the membrane potential change only adds very slightly to the computational cost. The other efficiency saving of a tau-leap style algorithm over channel-by channel calculation comes from treating a group of molecules (channels) as a single stochastic unit. On a neuron the unit size is determined by the electrical compartmentalization of the membrane. Therefore, depending on the geometry and channel distribution, clusters of up to 5000 channels may be possible. The channel update step yields conductances (assumed Ohmic) and reversal potentials for membrane currents on each compartment, which are used in the Crank-Nicolson or implicit Euler methods to compute new membrane potentials. The process is then repeated for the next step. The allocation of channels, discretization of the structure and tabulation of transition matrices is performed in a pre-processing stage written in Java. The core calculations are written in Fortran.

### Simulation of reconstructed neurons

For simulations illustrated in Figures 6–8 neuronal morphologies were downloaded from the Neuromorpho database (www.neuromorpho.org). Neurons were identified in the database as follows. Layer V pyramidal cells: p18 and p22 from Dendritica; g0692P1, g0699P1 and gR002P1 from Svoboda lab. Dentate gyrus granule cells: n271, n272 and n518 from Turner lab; 428883, B106885 from Claibourne lab. Purkinje cells: alxP, e4cb3a1 and e1cb4a1 from Martone lab; p19 and p20 from Dendritica. CA1 pyramidal cells: n409 from Turner lab; NM1 from Ascoli lab; ri04 and ri06 from Spruston lab; pc4c from Gulyas lab. Substantia nigra dopaminergic neurons: Nigra2a955, Nigra11h941-1, Nigra24a953, Nigra12h945 from Dendritica. Parvalbumin interneurons: pv08e, pv22b, pv22e, pv22j and pv22m from Gulyas lab. In these simulations the densities of voltage-gated channels were based on previously published studies [6]. The leak conductance was modeled as voltage-independent Na^+^ and K^+^ channels with open probabilities of 0.7. The following channels were included: fast Na^+^ channels (1/µm^2^); non-inactivating K^+^ channels (0.05/µm^2^); high-voltage Ca^2+^ channels (0.15/µm^2^); Na^+^ and K^+^ leak channels (0.016/µm^2^). The resting membrane potential was set by modifying the ratio of Na^+^ to K^+^ leak channels. In all simulations reported here this was −60 mV. We chose single-channel conductances of 20 pS for all ion channels, as this is similar to values reported for single channel recordings made from neuronal dendrites [54], [55]. This value is intermediate for cloned mammalian ion channels, which can have single channel conductances from <1 pS up to >150 pS [30]. Membrane capacitance was set to 0.75 µF/cm^2^ and axial resistivity to 150 Ω cm [6]. For models of neurons that are known to have dendritic spines (all models except the parvalbumin-expressing interneurons), the dendritic membrane capacitance and the number of dendritic ion channels were doubled. For each model neuron, membrane potential was recorded at the soma and at all dendritic locations 100 µm, 200 µm, 300 µm, etc., from the soma. All reported results were obtained from at least 3 s of simulated biological time. The simulation time step was 10 µs.

The simulations of a detailed model of CA1 pyramidal neuron (Figures 8 and 9) used previously published ion channels, morphology and channel distributions [44]. In this model voltage-dependent ion channels are distributed in the soma, axon and dendrites according to previous experimental measurements. The only modification to the model was the addition of *I*_h_ conductance and channel distribution taken from a different publication from the same group [45] and consistent with data from other groups [46]. The densities of Na^+^ and K^+^ leak channels were automatically adjusted to achieve a resting potential of approximately −70 mV throughout the cell, while maintaining a total leak conductance consistent with the original model. The single channel conductance of the delayed rectifier K^+^ channels and voltage-dependent Na^+^ channels were set to 20 pS, which is similar to estimates from single channel recordings [54], [55]. For simplicity, in the reported experiments the single channel conductance of A/D type K^+^ channels and leak channels were also set to 20 pS, which is similar to experimental measurements for D-type channels [55], somewhat larger than estimates for dendritic A-type channels [55] and towards the low end of the range of single-channel conductance reported for leak channels [30]. Thus, our simulations of models with only stochastically gating voltage-dependent Na^+^ and delayed rectifier K^+^ channels can be considered as fully constrained predictions given currently available data, while our simulations of the fully stochastic model likely estimate a lower limit for the consequences of stochastic ion channel gating. This is because our results from simulations when A/D type K^+^ channels are deterministic, but voltage-dependent Na^+^ or delayed rectifier K^+^ channels are stochastic, nevertheless demonstrate highly probabilistic spike firing, indicating that a smaller single channel conductance for A/D type K^+^ channels would have little impact on the results, while a possible larger single channel conductance for the leak channels would be expected to increase the impact of stochastic gating. Our simulations of A/D type stochastic gating alone should be considered as setting an upper limit for stochastic effects based on known properties of these channels, whereas the simulations of leak channels alone are less well constrained and serve as an illustrative example. Unlike other ion channels, the single channel conductance of *I*_h_ channels is set at 1 pS, which is consistent with noise-analysis of dendritic *I*_h_ recorded from cortical neurons [13] and the absence of step-like single channel waveforms from measurements of *I*_h_ obtained with cell-attached recordings from CA1 pyramidal neurons [46]. Synapses were modeled as bi-exponential conductance changes of rise time 0.2 ms, decay time 2 ms and peak conductance 0.18 nS. Synapses were distributed randomly across all dendrites >30 µm from the soma at an average density of 0.1/µm^2^ (1502 in total). Each synapse was activated independently according to a Poisson process with a mean frequency of 5.5 Hz. For analysis dendritic spike times were calculated as upward voltage crossings above a −60 mV threshold. Visual inspection of traces confirmed that this threshold successfully isolated all-or-nothing dendritic events.

Additional analysis of simulation data was carried out using IGORpro (Wavemetrics). Statistical analysis used R (http://www.r-project.org/). Comparisons of group data use ANOVAs. For analysis of data in Figures 8 and 9 this was followed by Tukey Honest Significant Difference test for comparisons between individual groups. Significance values referred to in the text refer to probabilities indicated by the latter test after adjustment for multiple comparisons. Simulations with NEURON used version 7.0 (www.neuron.yale.edu). For comparisons between NEURON and PSICS, simulations were run on the same hardware with minimal competing activity. Although other factors such as cache size and output options may contribute to performance differences in some cases, the simulation algorithm was the main factor determining simulation time. Parallel simulations were run on multi-processor PCs, or on a cluster of servers (maximum 1456 processors) at the Edinburgh Compute and Data Facility (ECDF).

## Supporting Information

Figure S1 Overview of PSICS. Model specification files are listed on a green background, simulation outputs on a yellow background and new software components on a clear background.
(0.93 MB JPG)

Figure S2 Sodium channel model. (A) The sodium channel model used to illustrate ion channel simulation with PSICS has a single open state (O1) connected as shown to two closed states (C1 and C2). (B) The transitions between states of the model are governed by forward α and backward β rate constants that vary as a function of membrane potential (upper graph). The time constants (Taux) and steady-state distribution (Xinf) for each transition are plotted as a function of membrane potential (lower graph). (C) Deterministic currents (bottom) generated by the gating scheme in response to step changes in membrane potential from a holding potential of 80 mV (top). Inset shows the activation phase of the currents on an expanded time base.
(0.25 MB JPG)

Figure S3 All-or-nothing dendritic responses of a fully stochastic CA1 pyramidal neuron model to synaptic stimulation. (A–B) Membrane potentials recordings from the soma (top) and indicated basal dendrite (bottom) from twenty consecutive trials as in Figure 8 and 9, illustrating responses to synaptic input corresponding to the time points in Figure 8D (A) and 8E (B). The fully propagating dendritic spike (A) and the smaller dendritic depolarizations (B) are all-or-nothing events, indicating that they result from triggering of dendritic spikes.
(0.24 MB TIF)

Figure S4 Synaptically driven spike output is modified by stochastic gating of single types of ion channel. Raster plots as in Figure 9, illustrating timing of action potentials generated in response to synaptic stimulation for versions of the CA1 pyramidal cell model in which the only stochastic gating ion channels are voltage-dependet Na^+^ channels (A), delayed rectifier K^+^ channels (B), A/D type K^+^ channels (C) and leak channels (D).
(0.50 MB JPG)

Text S1 Stochastic simulation framework; software development; estimate of the number of ion channels in a central neuron.
(0.05 MB PDF)
