# Supplementary figures and images for: Stochastic Ion Channel Gating in Dendritic Neurons: Morphology Dependence and Probabilistic Synaptic Activation of Dendritic Spikes
Source: PLoS Comput Biol. 2010 Aug 12;6(8):e1000886. doi: 10.1371/journal.pcbi.1000886 (PMC2920836; doi:10.1371/journal.pcbi.1000886)

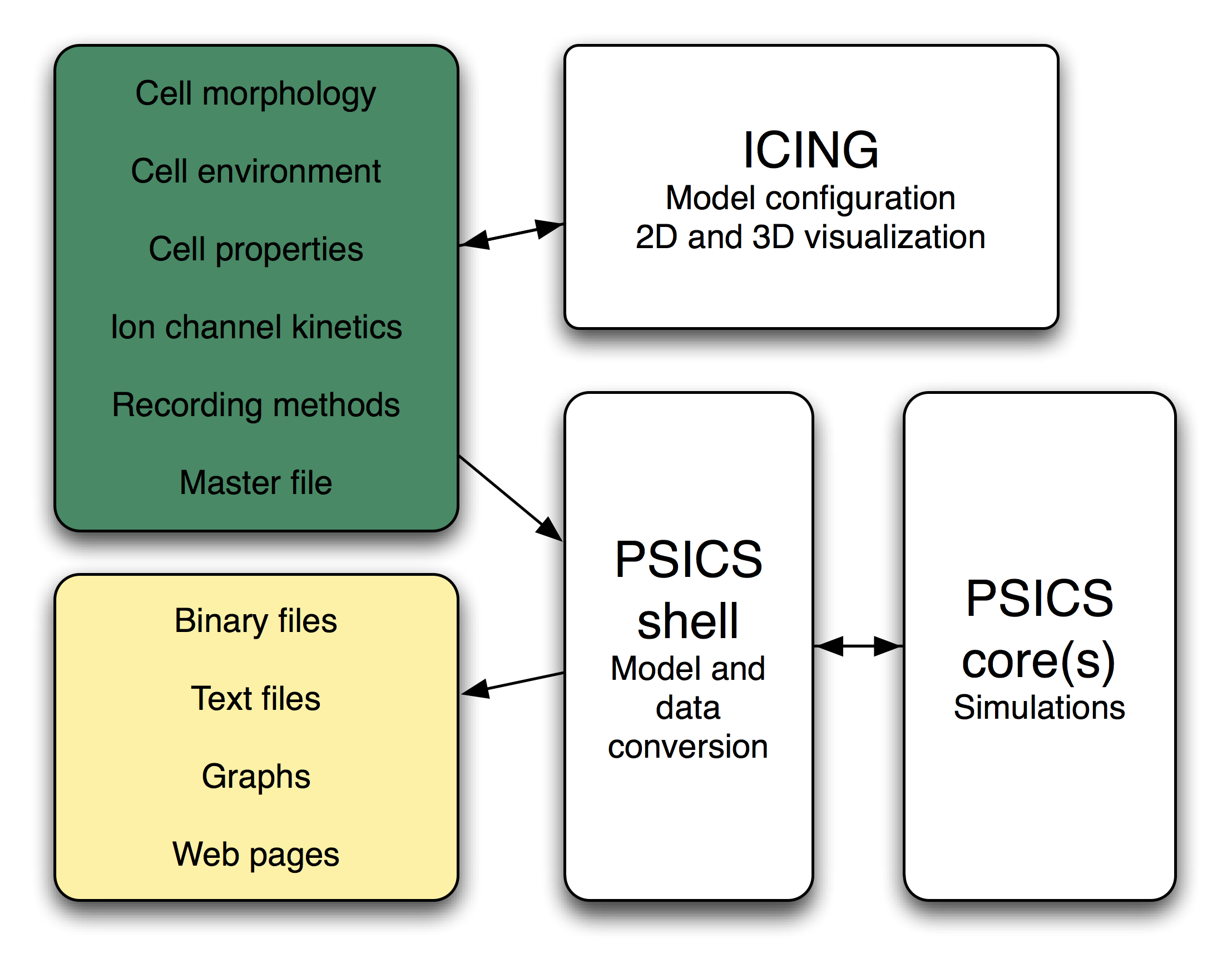

Supplement: Figure S1 — Overview of PSICS. Model specification files are listed on a green background, simulation outputs on a yellow background and new software components on a clear background. (0.93 MB JPG) [file pcbi.1000886.s001.jpg]

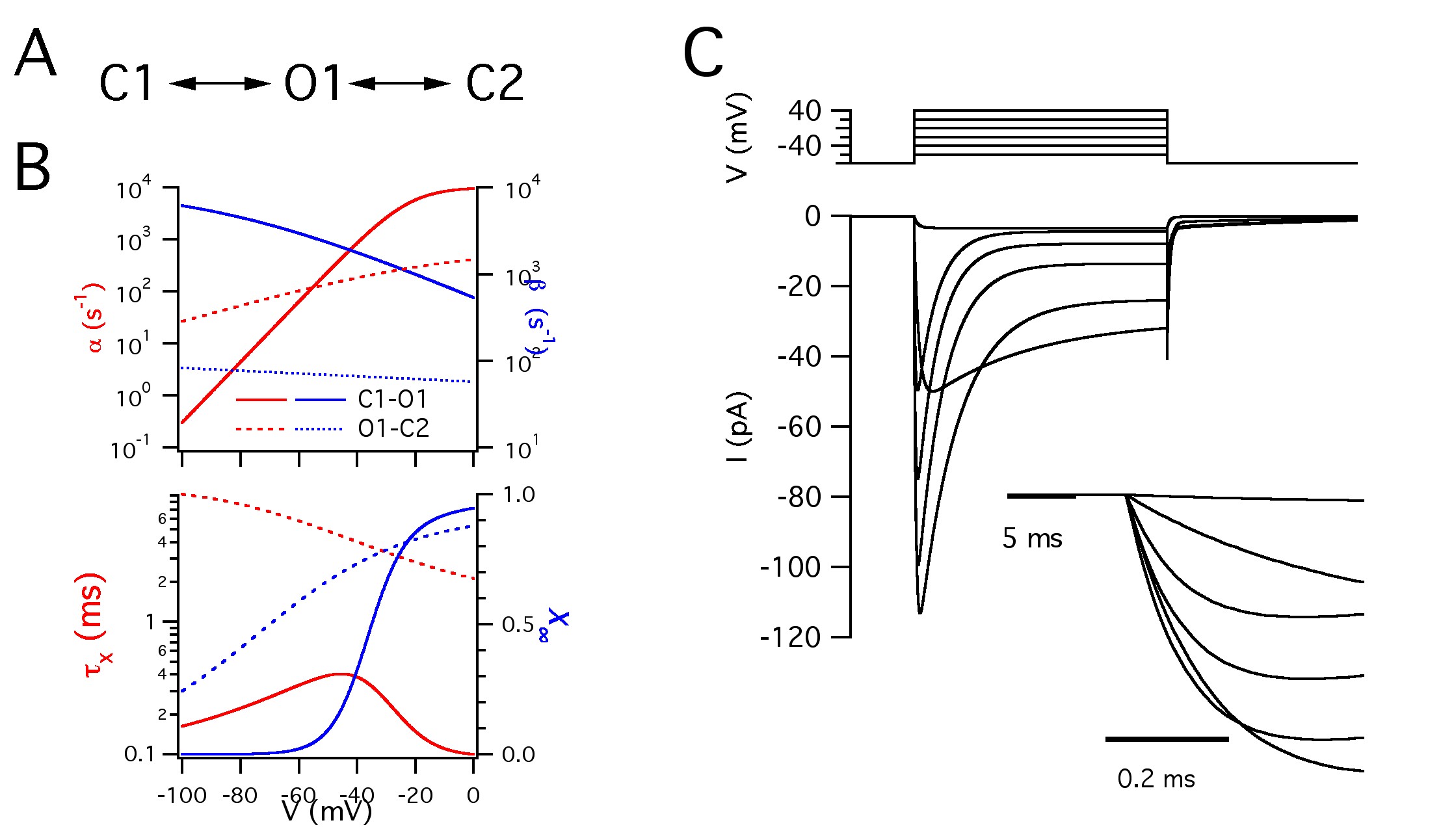

Supplement: Figure S2 — Sodium channel model. (A) The sodium channel model used to illustrate ion channel simulation with PSICS has a single open state (O1) connected as shown to two closed states (C1 and C2). (B) The transitions between states of the model are governed by forward α and backward β rate constants that vary as a function of membrane potential (upper graph). The time constants (Taux) and steady-state distribution (Xinf) for each transition are plotted as a function of membrane potential (lower graph). (C) Deterministic currents (bottom) generated by the gating scheme in response to step changes in membrane potential from a holding potential of 80 mV (top). Inset shows the activation phase of the currents on an expanded time base. (0.25 MB JPG) [file pcbi.1000886.s002.jpg]

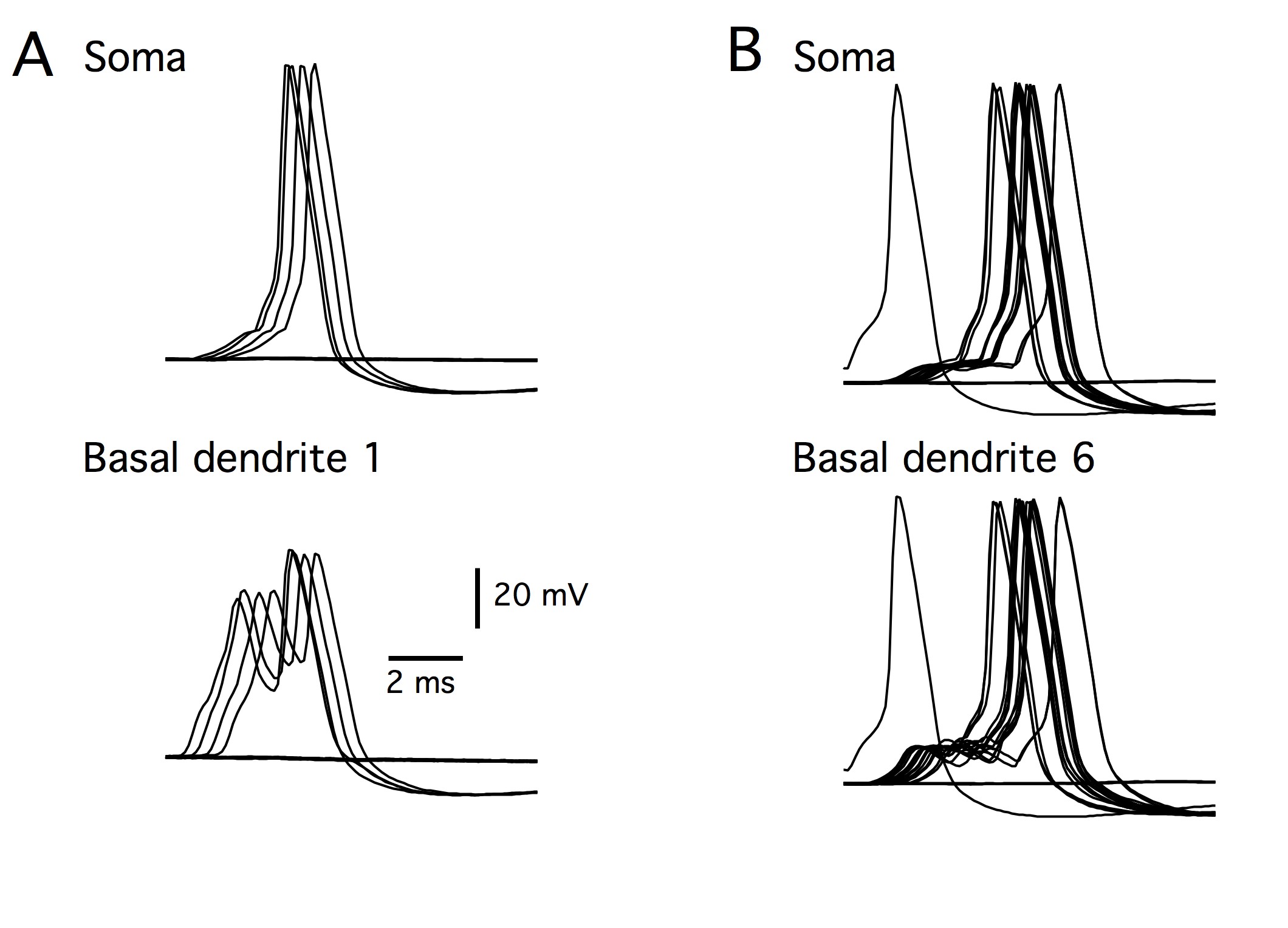

Supplement: Figure S3 — All-or-nothing dendritic responses of a fully stochastic CA1 pyramidal neuron model to synaptic stimulation. (A–B) Membrane potentials recordings from the soma (top) and indicated basal dendrite (bottom) from twenty consecutive trials as in Figure 8 and 9, illustrating responses to synaptic input corresponding to the time points in Figure 8D (A) and 8E (B). The fully propagating dendritic spike (A) and the smaller dendritic depolarizations (B) are all-or-nothing events, indicating that they result from triggering of dendritic spikes. (0.24 MB TIF) [file pcbi.1000886.s003.jpg]

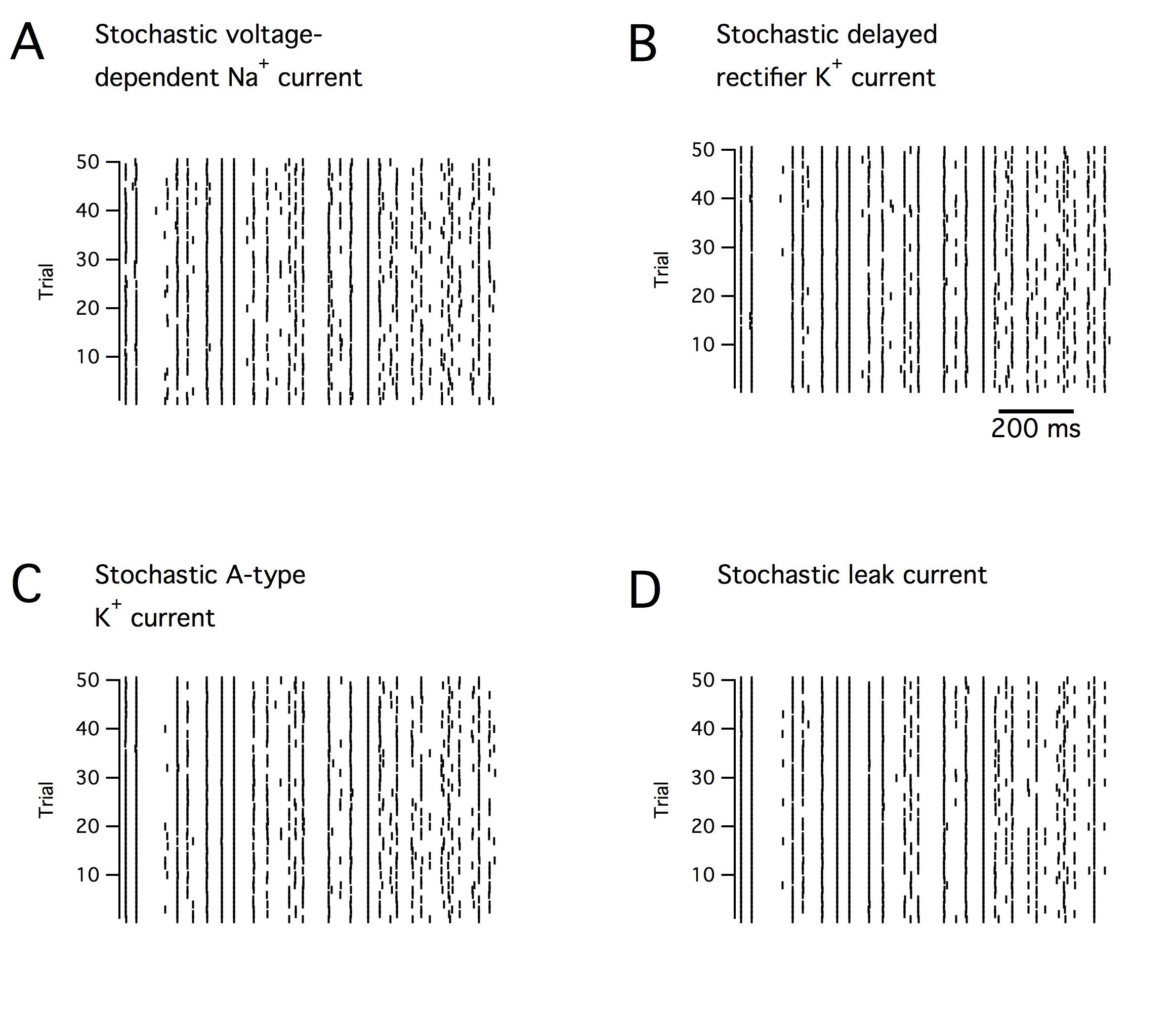

Supplement: Figure S4 — Synaptically driven spike output is modified by stochastic gating of single types of ion channel. Raster plots as in Figure 9, illustrating timing of action potentials generated in response to synaptic stimulation for versions of the CA1 pyramidal cell model in which the only stochastic gating ion channels are voltage-dependet Na+ channels (A), delayed rectifier K+ channels (B), A/D type K+ channels (C) and leak channels (D). (0.50 MB JPG) [file pcbi.1000886.s004.jpg]
