# Supplementary material for: Stochastic Ion Channel Gating in Dendritic Neurons: Morphology Dependence and Probabilistic Synaptic Activation of Dendritic Spikes
Source: PLoS Comput Biol. 2010 Aug 12;6(8):e1000886. doi: 10.1371/journal.pcbi.1000886 (PMC2920836; doi:10.1371/journal.pcbi.1000886)
Supplement: Text S1 — Stochastic simulation framework; software development; estimate of the number of ion channels in a central neuron. (0.05 MB PDF) [file pcbi.1000886.s005.pdf]

# Text S1

**Stochastic simulation framework.** Simulations in PSICS are carried out by an optimized inner core program which we describe below. The user specifies models and simulation controls in XML formatted files that are converted by a shell program into a form that can be handled efficiently in the computational core (Figure S1). Importantly, this strategy separates model specification from implementation of the simulation, enabling compatibility with other software and databases that use XML based standards for data sharing (Cannon et al., 2007; Crook et al., 2007; Goddard et al., 2001). The shell also formats output from simulations, for example to provide specified time series variables as binary files, text files, or as graphs, tables or text in html-formatted pages that can be displayed in a web browser. As the membranes of many neurons are likely to contain ¿ 106 ion channels (see Supplemental Text), tools for automatic generation of various distributions of ion channels are built into PSICS and so it is not necessary to manually specify the location of each ion channel.

To facilitate re-usability and reduce duplication of model components, PSICS simulations are specified by six stand-alone file types that are responsible for the following separate aspects of the model: cell morphology; cell environment; cell properties; ion channel kinetics; recording methods and a master file for overall specification of the simulation (Figure S1). Using a simple XML file structure enables components of a model either to be constructed manually, to be configured using ICING, or in the case of ion channels and morphologies to be imported from other programs and databases that allow saving of models in the NeuroML format.

**Software development.** There are three components to implementing rapid simulation of neurons with stochastic ion channels. First, it requires an efficient algorithm for the stochastic processes with a predictable operation count. This is described in the Experimental Procedures that accompany the main text. Second, it requires careful memory management to minimize cache misses. And, third, it requires all the other tasks required for the computation to be implemented sufficiently carefully that their total cost is small compared to the essential elements of the stochastic calculation.

In PSICS the following techniques have been used to meet these objectives:

1) Predefined scope and declarative model specification. A declarative specification sets firm boundaries on the models that can be run and makes the available structures explicit. The implementation can then use this information to convert models to their most efficient forms.

2) Preprocessing and tabulation. The model specification is processed independently of the main program into a compact specification for the calculation to be performed. User-defined functions are evaluated in advance on a grid. The core calculation only interpolates in this grid for transition rates etc, and is therefore independent of the functional forms used in the model.

3) Language separation. The preprocessing stage is not time critical and can be implemented in a convenient object orientated language (Java is used for PSICS). For the core calculation the focus is on memory management and operation counting so a language developed for high performance computing is preferable (Fortran is used for PSICS).

4) Isolating core calculations. The use of tabulation and interpolation throughout means that all calculations have the same structure which, in turn, reduces the length of the core code and facilitates good memory management and profiling.

5) Profiling tools, such as Valgrind (http://valgrind.org) can be used to monitor cache misses and other computational costs to locate aspects that can be improved. Having a concise, easily costed core algorithm, sets a scale for comparing other parts of the calculation and avoiding unnecessary optimization effort.

6) Code optimization. The above techniques lead to very concise core calculations (most of the time is spent in the same 100 lines) which can then be experimentally tuned with regard to the algorithm, such as the trade-offs between accuracy in fitting functions as in section xxx and the extra costs of evaluating powers or logarithms.

**Estimate of the number of ion channels in a central neuron.** We assume that a rodent pyramidal neuron has a membrane surface area of that may be greater than 60,000 m2, but more

typically is approximately 35,000 m2 (Scorcioni et al., 2004). We also assume that non-synaptic ion channels are distributed throughout a neurons soma and dendrites at densities of up to 1000 ion channels m-2 (e.g. (Engel and Jonas, 2005; Kole et al., 2006)). This suggests that the membrane of a typical neuron may contain up to 107, but perhaps more conservatively on the order of 106 individual non-synaptic ion channels.

## References


Cannon, R.C., Gewaltig, M.O., Gleeson, P., Bhalla, U.S., Cornelis, H., Hines, M.L., Howell, F.W., Muller, E., Stiles, J.R., Wils, S., et al. (2007). Interoperability of neuroscience modeling software: current status and future directions. Neuroinformatics 5, 127-138.

Crook, S., Gleeson, P., Howell, F., Svitak, J., and Silver, R.A. (2007). MorphML: level 1 of the NeuroML standards for neuronal morphology data and model specification. Neuroinformatics 5, 96-104.

Engel, D., and Jonas, P. (2005). Presynaptic action potential amplification by voltage-gated Na+ channels in hippocampal mossy fiber boutons. Neuron 45, 405-417.

Goddard, N.H., Hucka, M., Howell, F., Cornelis, H., Shankar, K., and Beeman, D. (2001). Towards NeuroML: model description methods for collaborative modelling in neuroscience. Philos Trans R Soc Lond B Biol Sci 356, 1209-1228.

Kole, M.H., Hallermann, S., and Stuart, G.J. (2006). Single Ih channels in pyramidal neuron dendrites: properties, distribution, and impact on action potential output. J Neurosci 26, 1677-1687.

Scorcioni, R., Lazarewicz, M.T., and Ascoli, G.A. (2004). Quantitative morphometry of hippocampal pyramidal cells: differences between anatomical classes and reconstructing laboratories. J Comp Neurol 473, 177-193.
